# Extranasopharyngeal angiofibroma of the nasal septum - uncommon presentation of a rare disease

**DOI:** 10.5935/1808-8694.20130118

**Published:** 2015-10-08

**Authors:** Felipe Gustavo Correia, Juliana Caminha Simões, José Arruda Mendes-Neto, Maria Teresa de Seixas-Alves, Luis Carlos Gregório, Eduardo Macoto Kosugi

**Affiliations:** a MD. Graduated from the Paulista School of Medicine - Federal University of São Paulo - UNIFESP - EPM.; b MD. Graduated from the Federal University of Ceará; Second-Year Medical Resident in Otolaryngology - UNIFESP - EPM.; c MSc in Sciences - UNIFESP-EPM; Assistant Physician at the Rhinology Unit - UNIFESP - EPM.; d Senior Associate Professor; Head of the Pathology Department - UNIFESP - EPM.; e PhD in Sciences - UNIFESP-EPM; Deputy Head of the Department of Otolaryngology and Head and Neck Surgery - UNIFESP-EPM.; f PhD in Sciences - UNIFESP-EPM; Coordinator of the Fellowship in Rhinology Program - UNIFESP-EPM; Chief Preceptor of the Residency Program in Otolaryngology - UNIFESP-EPM. Rhinology Division - Department of Otorhinolaryngology and Head and Neck Surgery - Paulista School of Medicine - Federal University of São Paulo.

**Keywords:** angiofibroma, differential diagnosis, nasal septum

## INTRODUCTION

The extranasopharyngeal angiofibroma (ENPA) is a tumor which is histologically similar to juvenile nasopharyngeal angiofibroma (JNA), differing from the latter in clinical and epidemiological characteristics^1^, ^2^, ^3^. Prevalence, gender, age, affected site, pathogenesis, clinical-CT and recurrence are completely different^1^, ^2^, ^3^, which leads some authors to classify the ENPA as a disease which is different from the JNA^1^.

There are less than a hundred cases of ENPA described in the literature, and the maxillary sinus is the most frequently affected site, followed by the ethmoid, being rare in the nasal septum^1^, ^2^, ^3^. The objective of this study is to report a case of ENPA with a rare presentation in the nasal septum.

## CASE PRESENTATION

**Figure 1:**
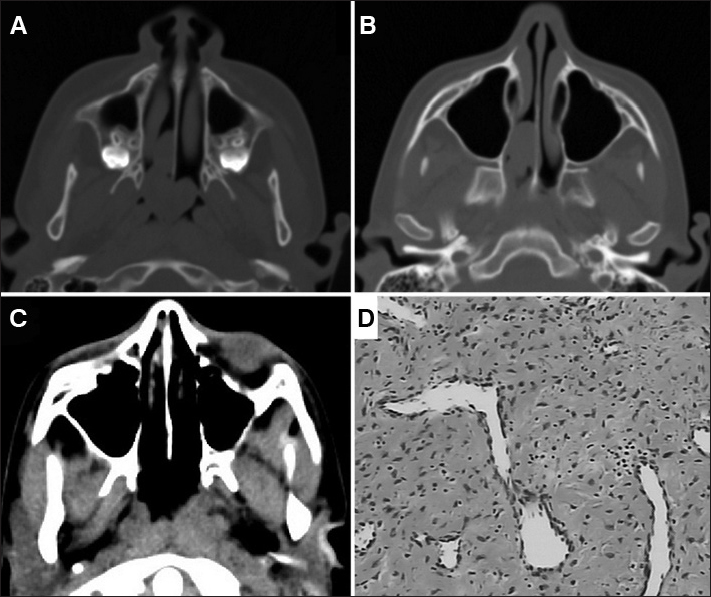
A: Preoperative axial CT scan showing the tumor inserted into the nasal septum and extending to the choanae; B: Axial CT scan showing preoperative pterygopalatine fossa without disease involvement; C: Axial CT scan postoperatively; D: HE histological section showing spindle cell proliferation with hyalinization areas intermingled with vessels - sometimes arched.

WSR, 10 years and 11 months of age, complained of constant bilateral nasal obstruction for six months, worse on the right side, with hyposmia and snoring, without epistaxis. Nasal endoscopy showed a pinkish lesion, smooth, non-friable, non-ulcerated, apparently inserted into the nasal septum, obstructing the right nasal cavity (RNC) in its posterior third all the way to the choana, and it could be viewed through the other nostril. Middle meatuses and sphenoethmoidal recesses were free. Computed tomography (CT) showed a lesion with soft tissue density in the RNC (Figure 1A-B). We performed an endoscopic approach, identifying the lesion inserted in the nasal septum, doing a subperiosteal dissection and excision with a margin at its insertion. Postoperative follow-up of 1 year and 9 months without recurrence (Figure 1C). Histopathology reported it to be an angiofibroma (Figure 1D).

## DISCUSSION

The JNA is the most common benign neoplasm of the nasopharynx, despite representing less than 0.05% of tumors of the head and neck^1^, ^2^, ^4^, ^5^. It affects almost exclusively males, between 12 and 14 years of age^1^, ^2^, ^4^, ^5^. But the ENPA is even more unusual, more common in women between 17 and 22 years, and its most common site is the maxillary sinus, followed by the ethmoid, being very rare in the nasal septum and inferior turbinates^1^, ^2^, ^3^. The origin of the JNA is at the top of the sphenopalatine foramen^1^, ^2^, ^5^ with controversial etiology^4^. ENPA's etiology is associated with a migration error of the fascia basalis^1^, justifying its presence in varied locations^2^. Our patient had age, gender and location different from most ENPAs, confirming the rarity of this case.

The initial growth of the JNA follows a well-defined pattern in the nasal cavity, nasopharynx and pterygopalatine fossa^4^, leading to the triad: nasal obstruction, recurrent epistaxis and nasopharyngeal tumor^1^, ^2^, ^5^. The JNA has characteristic radiological signs: Holman-Miller (anteriorization of the posterior wall of the maxillary sinus) and enlargement of the sphenopalatine foramen - pterygopalatine fossa^1^, ^2^, ^4^, ^5^. The ENPA can evolve with a variety of symptoms and radiological signs, depending on its site^1^, ^2^. Our patient reported nasal obstruction due to a rare location in the nasal septum.

Histologically, the ENPA is similar to the JNA, with connective tissue stroma and a matrix of dilated vessels without a muscular layer^2^, ^3^, ^5^. As for differential diagnosis, we have the hemangioma and the hemangiopericitoma^3^. While the JNA can be suspected based on known clinical and CT chracteristics^2^, ^4^, ^5^, histopathological examination is essential to confirm the ENPA diagnosis^1^. Treatment is surgical in both diseases^2^. Although the ENPA is nurtured by the maxillary artery^4^ (just like the JNA), it may not yield excessive intraoperative bleeding due to the predominance of fibrous stroma, unlike the JNA^1^, ^2^. Although benign, the JNA is locally aggressive, with recurrence rates of 6% to 27.5%^2^ due to incomplete tumor removal^5^. The ENPA usually does not recur because its extrapharyngeal location facilitates total resection^1^, ^2^, ^3^. Our patient did not complain of epistaxis, had no excessive intraoperative bleeding and is now 1 year and 9 months without recurrence.

Therefore, although histologically similar, the ENPA and the JNA may be considered different diseases, due to totally different pathogenesis, epidemiology, clinical and tomographic presentations^1^.

## FINAL REMARKS

Although rare, the ENPA should be considered in the diagnosis of vascular tumors of the head and neck. ENPA's clinical and epidemiological characteristics are different from those of the JNA.
